# Supplementary material for: Highly parallel and efficient single cell mRNA sequencing with paired picoliter chambers
Source: Nat Commun. 2020 Apr 30;11:2118. doi: 10.1038/s41467-020-15765-0 (PMC7193604; doi:10.1038/s41467-020-15765-0)
Supplement: Supplementary file 1 — Description of Additional Supplementary Files [file 41467_2020_15765_MOESM1_ESM.pdf]

Title: Supplementary Data1.

Description: Differential gene expression of mES cells

Title: Supplementary Data 2.

Description: Differential gene expression of drug treated and untreated cells

Title: Supplementary Movie 1.

Description: Paired-seq operation on chip

Title: Supplementary Movie 2.

Description: Function of control layer

Title: Supplementary Movie 3.

Description: Paired unit isolation

Title: Supplementary Movie 4.

Description: Beads capture-1 row

Title: Supplementary Movie 5.

Description: Cell capture

Title: Supplementary Movie 6.

Description: Blocking effect

Title: Supplementary Movie 7.

Description: Mixing

Title: Supplementary Movie 8.

Description: Single cell lysis
