# Supplementary material for: Highly parallel and efficient single cell mRNA sequencing with paired picoliter chambers
Source: Nat Commun. 2020 Apr 30;11:2118. doi: 10.1038/s41467-020-15765-0 (PMC7193604; doi:10.1038/s41467-020-15765-0)
Supplement: Supplementary file 2 — Supplementary Information [file 41467_2020_15765_MOESM2_ESM.pdf]

Highly Parallel and Efficient Single Cell mRNA Sequencing with Paired Picoliter Chambers

Zhang et al.

Highly Parallel and Efficient Single Cell mRNA Sequencing with Paired Picoliter Chambers

Mingxia Zhang[1*], Yuan Zou[1,2*], Xing Xu[1*], Xuebing Zhang[5], Mingxuan Gao[1], Jia Song[3], Peifeng Huang[1], Qin Chen[5], Zhi Zhu[1], Wei Lin[4], Richard N. Zare[2], Chaoyong Yang[1,3†]

Table of Contents

A. Supplementary Figure Legends

Supplementary Figure 1| Schematics of Paired-seq chip

Supplementary Figure 2| Characterization of structure and sequencing performance of Paired-seq chip with 2000 units

Supplementary Figure 3| Fabrication of Paired-seq chip

Supplementary Figure 4| Cell occupancy ratio on Paired-seq chip with different cell sizes

Supplementary Figure 5| Beads recovery from Paired-seq chip

Supplementary Figure 6| Isolation level of blocking valve and the cleaning effect

Supplementary Figure 7| Paired droplets mixing by free diffusion and pump driving on chip

Supplementary Figure 8| Single cell lysis on chip

Supplementary Figure 9| Comparing the mRNA expression levels with different loading time on chip

Supplementary Figure 10| Unique Molecular Identifier Filtering and Doublet rate

Supplementary Figure 11| Comparing the reads mapping quality of Exonuclease I treatment on chip and in tube

Supplementary Figure 12| Experiment design and data analysis for characterization of sequencing ability

Supplementary Figure 13| Accuracy of different mRNA sequencing methods through ERCC detection

Supplementary Figure 14| Sensitivity of different mRNA sequencing methods through ERCC detection

Supplementary Figure 15| Performance metrics in consideration of sequencing depth

Supplementary Figure 16| Reads mapping quality in mES cells cultured with different days after LIF withdrawal

Supplementary Figure 17| Parameters for t-SNE of mES cells

Supplementary Figure 18| Single cell trajectories of differentiated mES cells

Supplementary Figure 19| Nocodazole treatment of K562

Supplementary Figure 20| Reads mapping quality in Nocodazole treated and untreated K562 cells

Supplementary Figure 21| Schematic diagram for theoretical calculation of fluid resistance in chip channel.

B. Supplementary Table Legends

Supplementary Table 1. Geometric dimensions of paired unit.

Supplementary Table 2. Oligos used in Paired-seq

Supplementary Table 3. Differential gene expression of mES cells

Supplementary Table 4. Differential gene expression of drug treatment

Supplementary Table 5. Data source published on line for platform comparison

C. Step by step protocol for Paired-seq

1. Reagents

2. Primers
3. Consumables
4. Special equipment
5. Protocol

D. Supplemental References

A. Supplementary Figure Legends

A

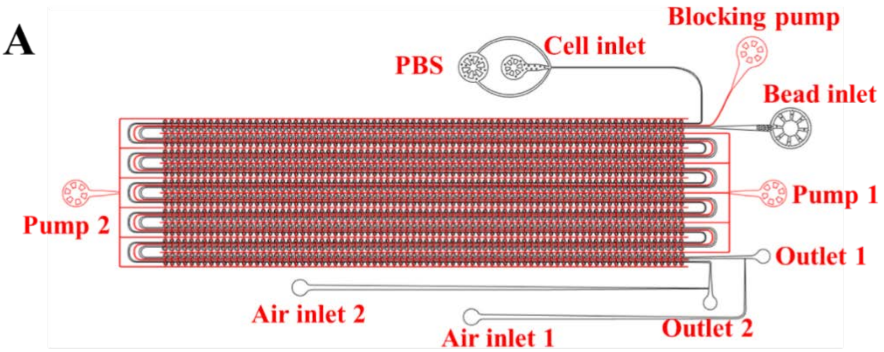

B

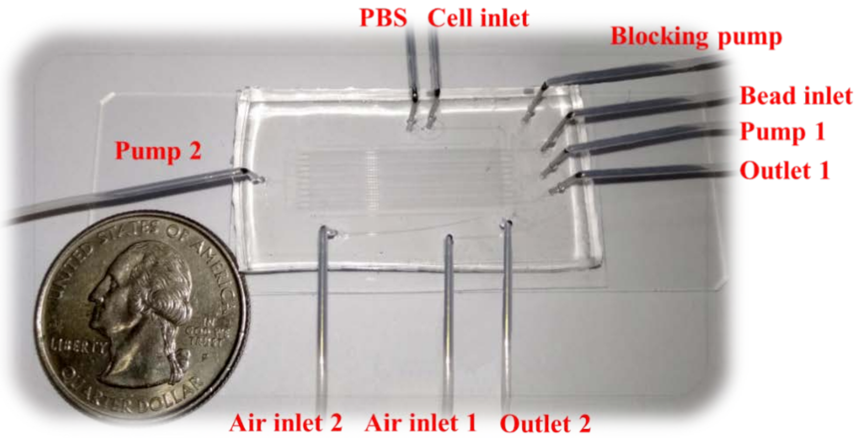

Supplementary Figure 1| Schematics of Paired-seq chip

(A) The CAD design and (B) actual chip with 800 pairing units. Capture layer (black) has three inlets for beads, cells and PBS buffer, respectively and two inlets for air. Control layer (red) has inlets for blocking valve, bead driving pump (pump 1) and cell driving pump (pump 2).

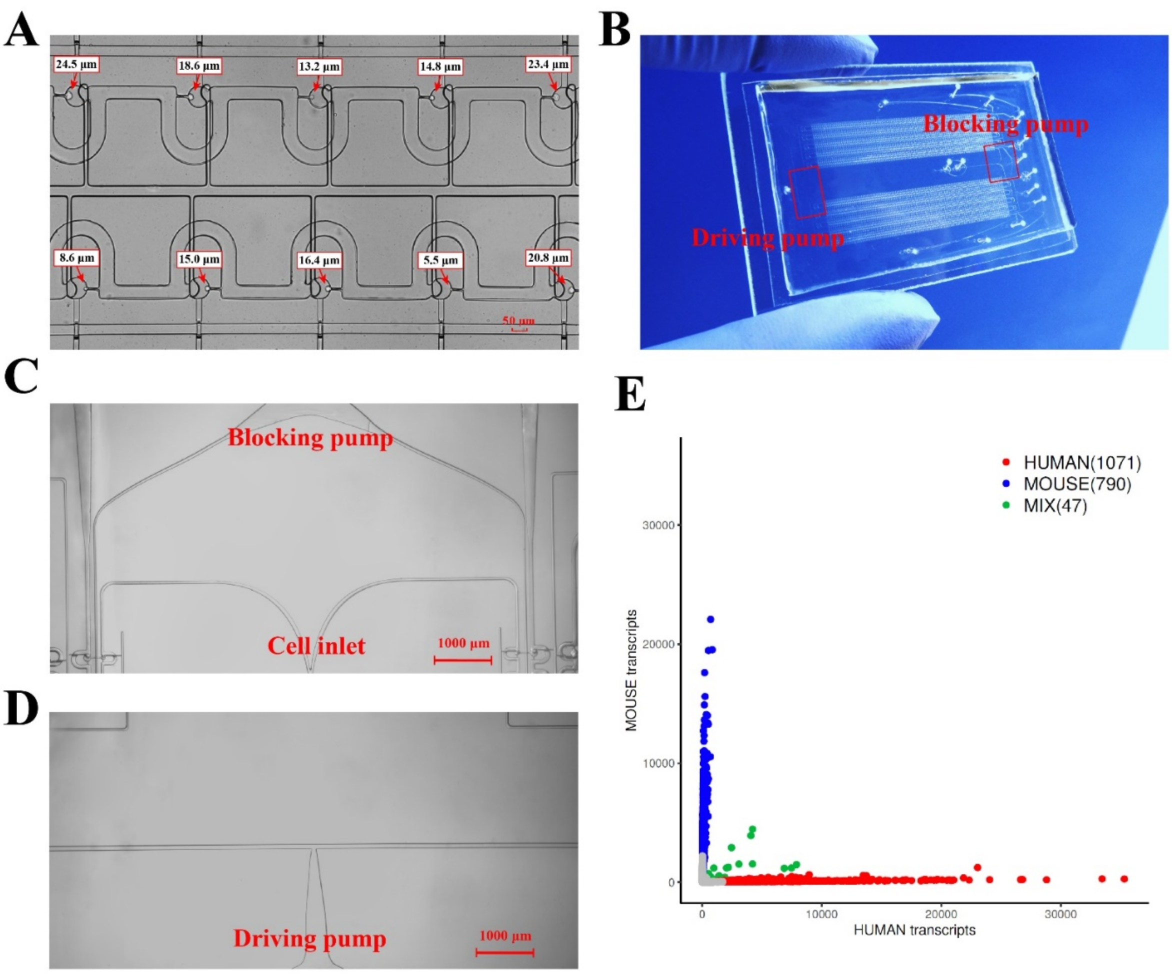

Supplementary Figure 2| Characterization of structure and sequencing performance of Paired-seq chip with 2000 units.

(A) Cell captured on Paired-seq chip with 2000 units.

(B) Photograph of Paired-seq chip with 2000 units.

(C) The design of driving pump for Paired-seq chip with 2000 units.

(D) The design of blocking pump and cell inlet for Paired-seq chip with 2000 units.

(E) Human-mouse mixture experiment using Paired-seq with 2000 units.

Source data are provided as a Source Data file.

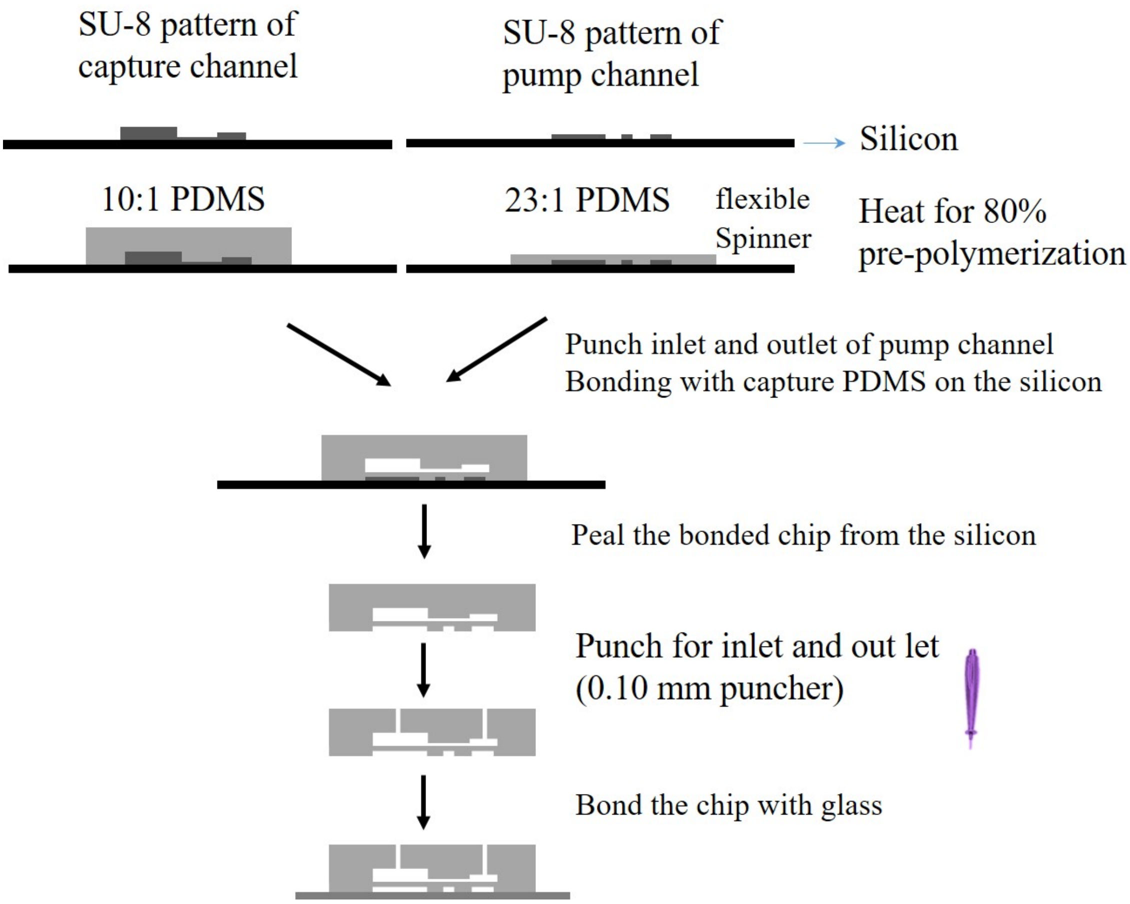

Supplementary Figure 3| Fabrication of Paired-seq chip.

We have described the details of chip fabrication at Methods in the main text.

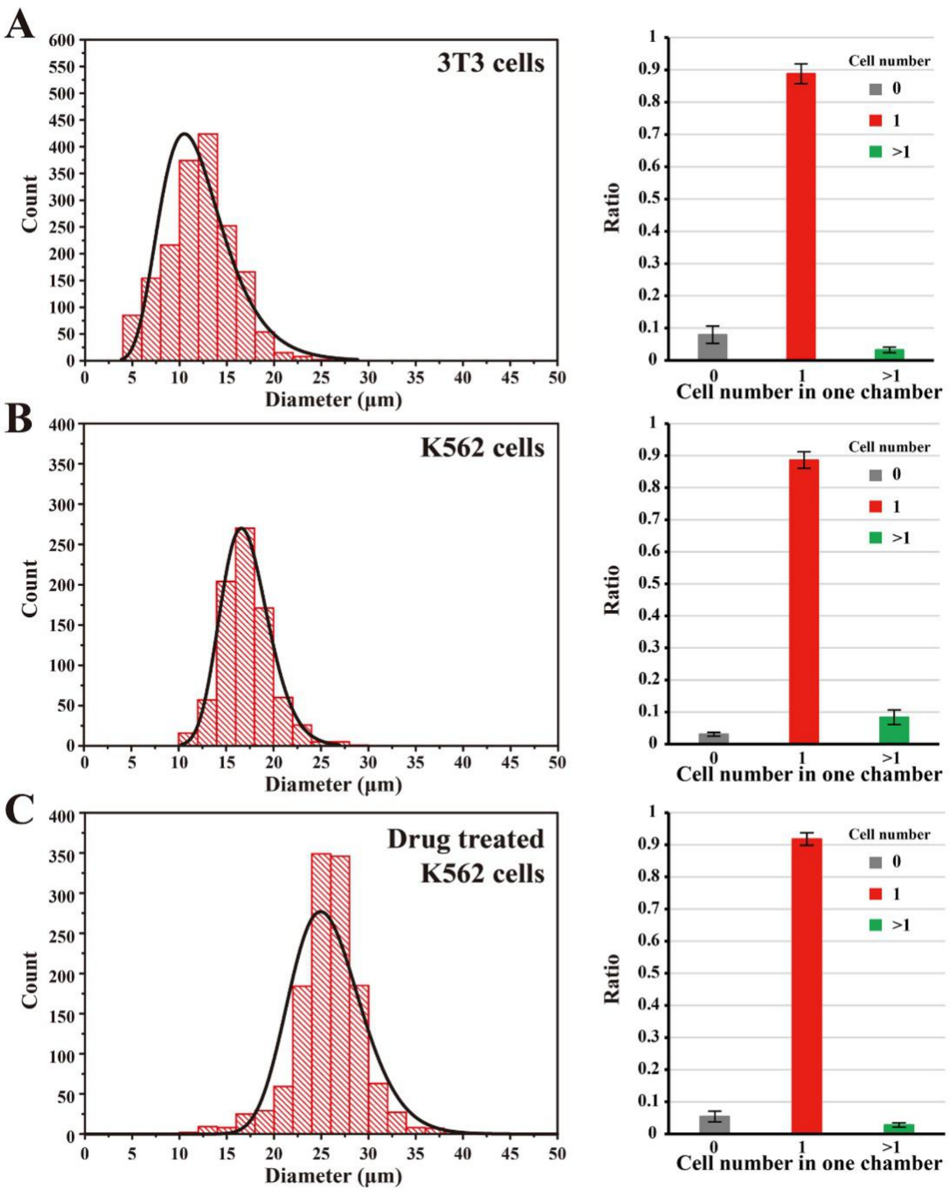

Supplementary Figure 4| Cell occupancy ratio on Paired-seq chip with different cell sizes.

To test the influence of cell size on occupancy ratio, we performed cell capture on Paired-seq chip with different cell sizes and calculated the occupancy ratio.

A. Diameter of small size cell line (left) and the occupancy ratio. Cell line: 3T3. Error bars, mean ± s.d., n = 3.

B. Diameter of medium size cell line (left) and the occupancy ratio. Cell line: K562. Error bars, mean ± s.d., n = 3.

C. Diameter of large size cells (left) and the occupancy ratio. Cell: drug treated (*Nocodazole*) K562 cells. Error bars, mean ± s.d., n = 3.

Source data are provided as a Source Data file.

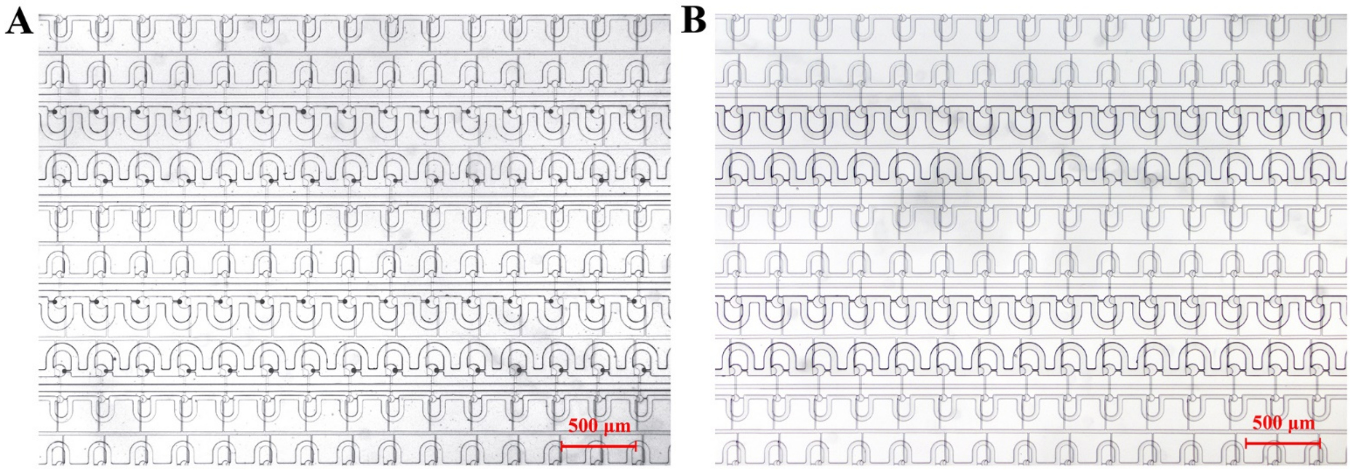

Supplementary Figure 5| Beads recovery from Paired-seq chip

The barcoded beads with cDNA information from individual cells are expelled out of the capture chamber with a flow of solution in the reverse direction. Chip image before (A) and after (B) bead recovery.

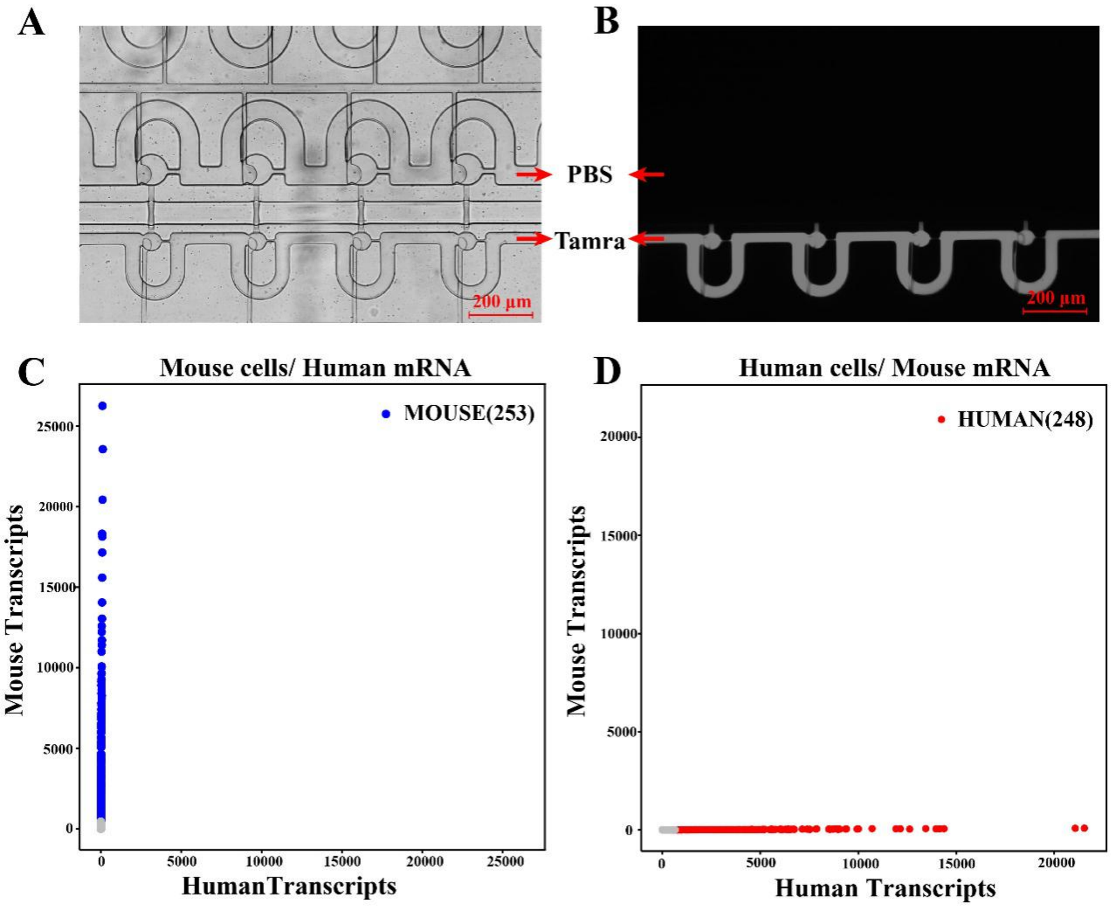

Supplementary Figure 6| Leakage test of blocking valve and cell-free mRNAs removal capability

(A)(B) The fluorescence image monitoring exchange and diffusion of small fluorescent molecules with the blocking valve on. Scale bar is 200 µm. In A, B, representative results from more than three independent experiments are shown.

(C)(D) The sequencing result inspecting contamination of cell-free mRNAs in Paired-seq, which reflects the isolation level of the blocking valve and the cleaning effect.

Source data are provided as a Source Data file.

A

B

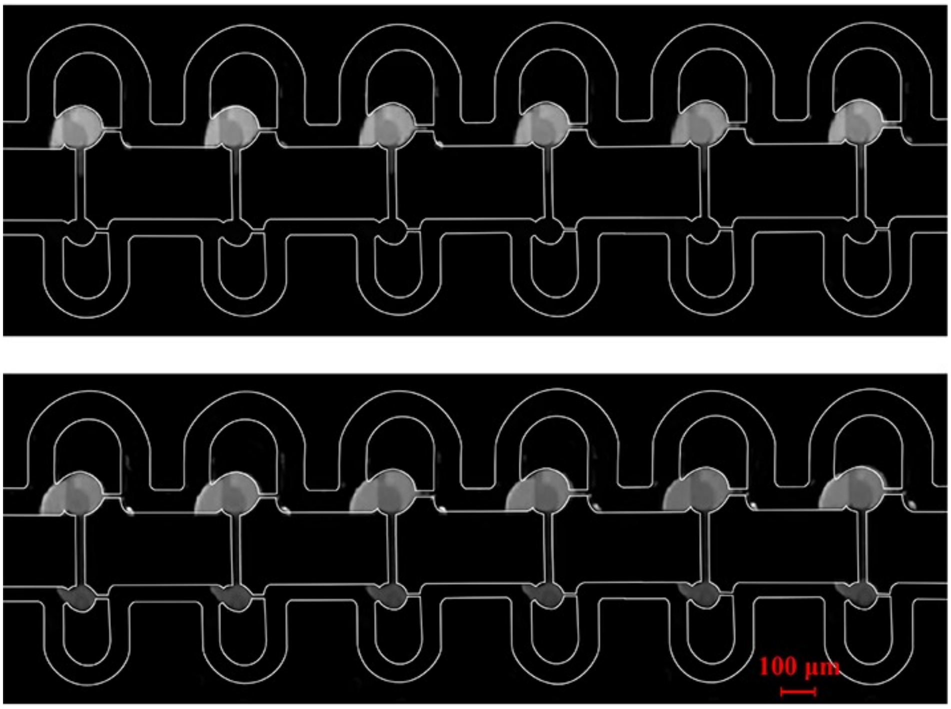

Supplementary Figure 7| Paired droplets mixing by free diffusion and pump driving on chip

(A). Paired droplets mixing by free diffusion for 40 s. Scale bar is 100 µm.

(B). Paired droplets mixing by pump driving for 10 s. Scale bar is 100 µm.

In A, B, representative results from more than three independent experiments are shown.

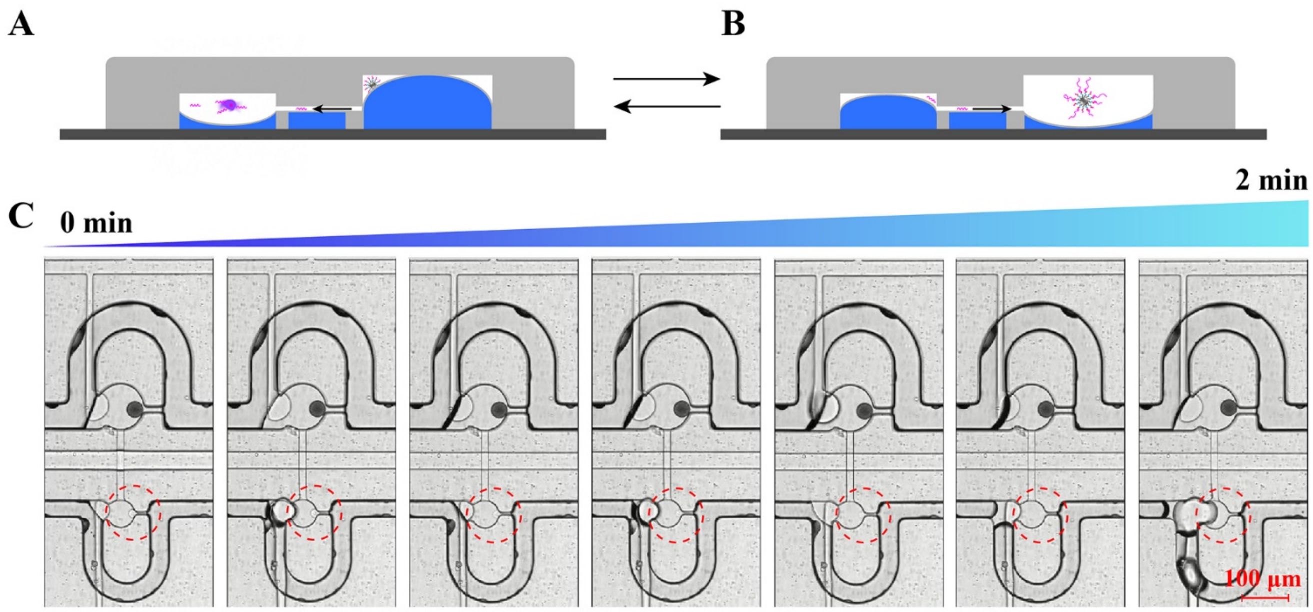

Supplementary Figure 8| Single cell lysis on chip

(A) With the help of two driving pumps, lysis buffer can be efficiently delivered to the cell chamber in order to achieve complete cell lysis and maximum mRNAs captured by beads (B).

(C) Cell lysis on chip with the blocking valve off. Scale bar is 100 μm. Cell number >100.

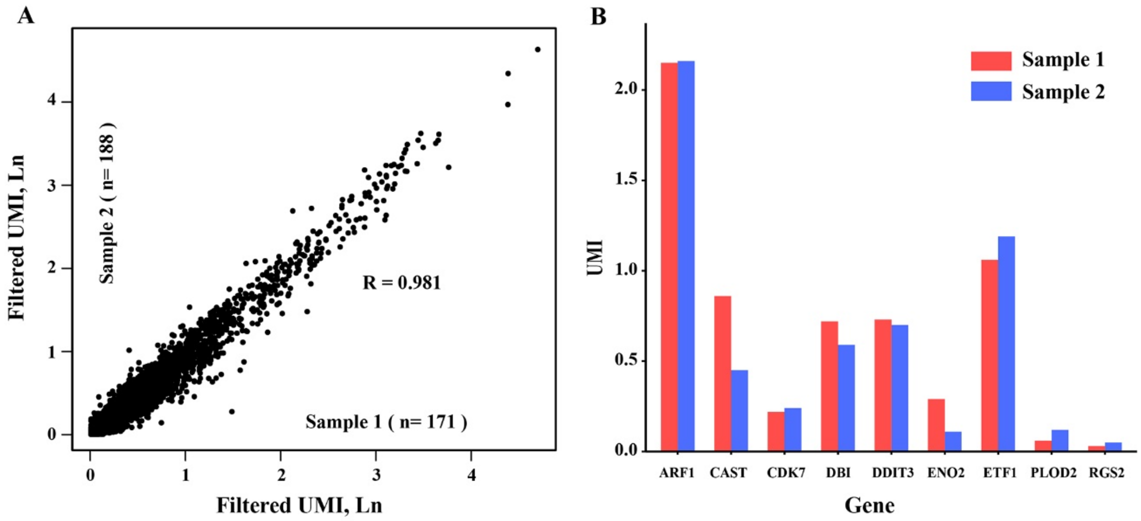

Supplementary Figure 9| Comparing the mRNA expression levels with different washing time on chip. To test the influence of shear forces on RNA quality or transcription, we compared the gene detection ability reflecting RNA integrity with different loading time. Our results showed that at the loading time of 15 min (sample 1) and 40 min (sample 2), the number of detected genes show no significant difference (A). We also analyzed the expression levels of 9 genes (*ARF1*, *CAST*, *CDK7*, *DBI*, *DDIT3*, *ENO2*, *ETF1*, *PLOD*2 and *RGS2*) reported to have correlations with mechanical stress[1]. Herein, the 9 genes were biologically well characterized in terms of protein function, including cell communication, cell signaling, cell cycle, stress response and calcium release. The result show no remarkable differences between the samples with different loading time, indicating that the shear force did no damage to the cells. This data suggest that long loading time was not detrimental to RNA quality or spurious/stress related transcription. Source data are provided as a Source Data file.

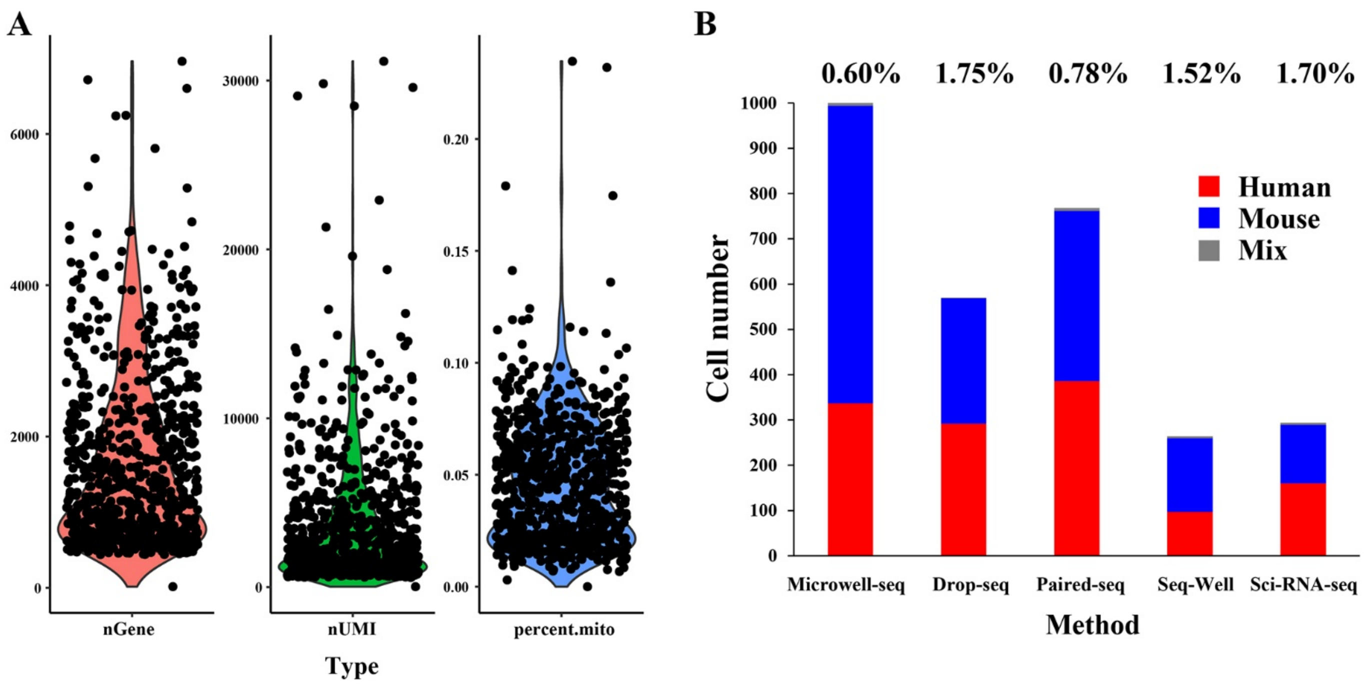

Supplementary Figure 10| Unique Molecular Identifier Filtering and Doublet rate.

(A) Reads mapping quality in human-mouse mixture experiment.

(B) Doublet rate comparison of mixed-species experiment with different platforms.

Source data are provided as a Source Data file.

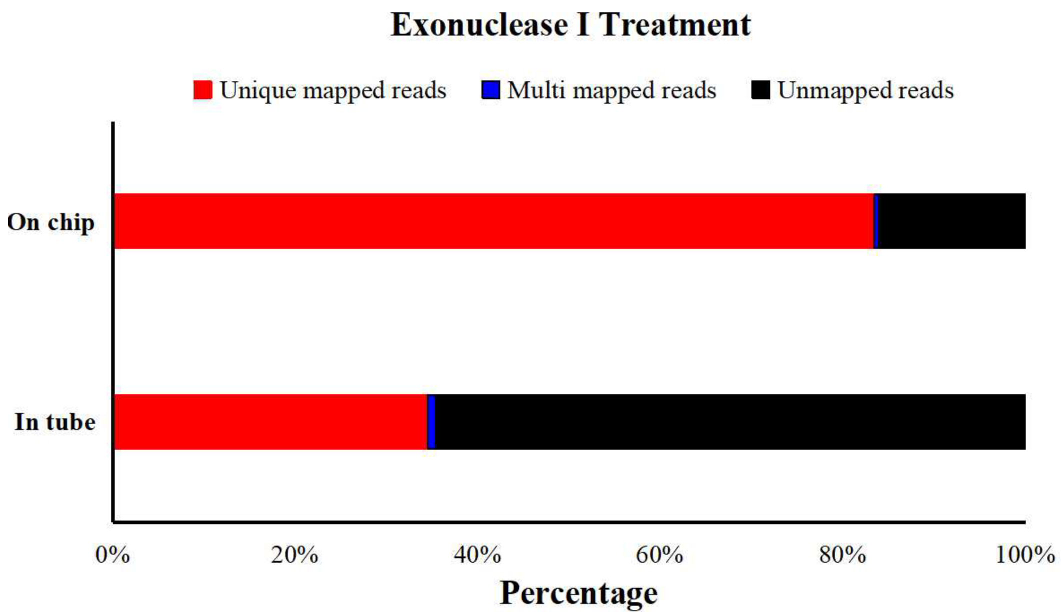

Supplementary Figure 11| Comparing the reads mapping quality of Exonuclease I treatment on chip and in tube.

In order to optimize the experimental operation and check if the integrated of enzyme reactions on a chip, we compared the ERCC experiment by using Paired-seq with enzymatic process on and off chip (in tube). The results showed that the percentages of mapped reads for enzymatic process on chip were significantly higher than that in tube. Most of the unmapped reads (due to too short sequence) in off-chip sample could be traced back to primer on the barcoded beads, which confirmed that the insufficient enzymatic reaction brings in technical noise.

Source data are provided as a Source Data file.

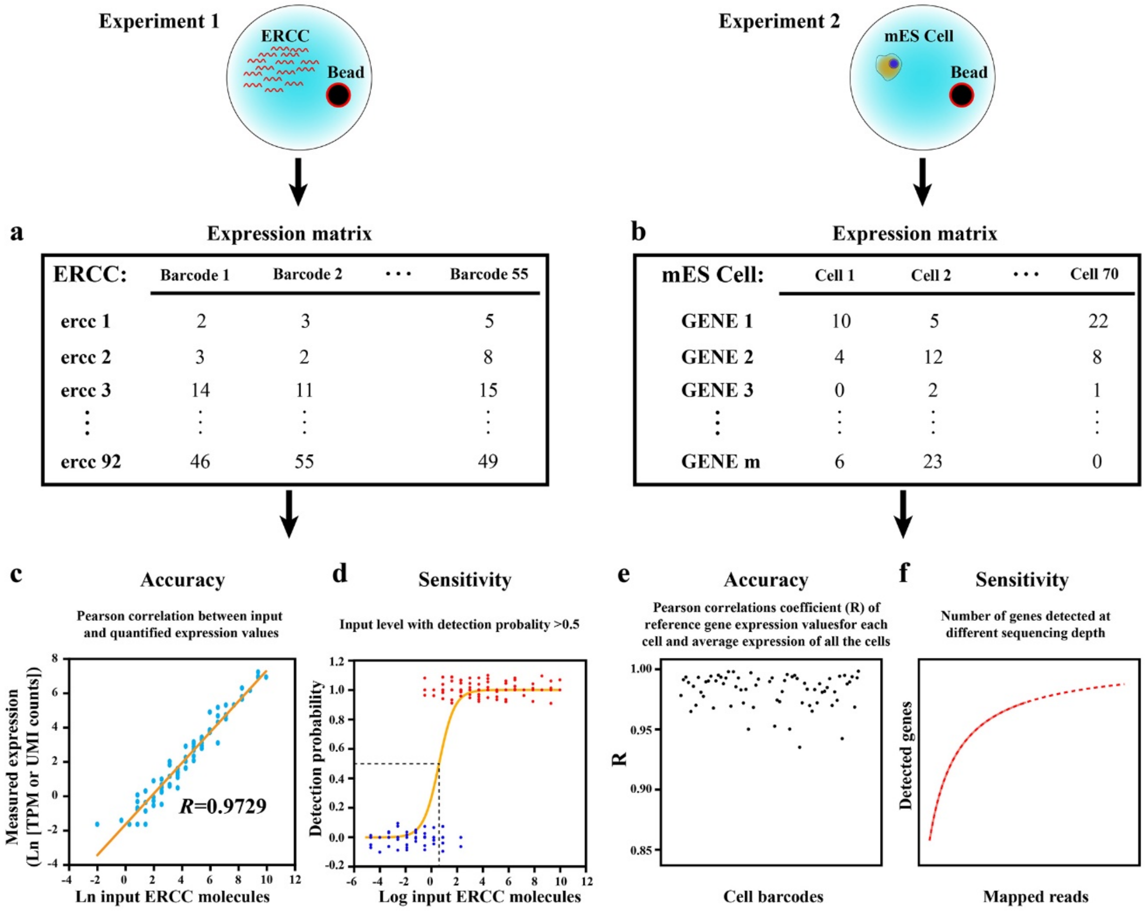

Supplementary Figure 12| Experimental and computational workflow for accuracy and sensitivity characterization

(a). Expression matrix of ERCC barcoded beads.

(b). Expression matrix of mES cells.

(c). Accuracy of scRNA-seq with ERCC.

(d). Sensitivity of scRNA-seq with ERCC.

(e). Accuracy of scRNA-seq with mES cells. Pearson correlation coefficient (R) of reference gene expression values for each cell and average expression of all the cells were calculated and shown in the plot. Each dot represents a cell.

(f). Sensitivity of scRNA-seq with mES cells. Fitted (solid line) and predicted (dashed line) curve of median genes detected in a single mES cell versus varying mapped reads.

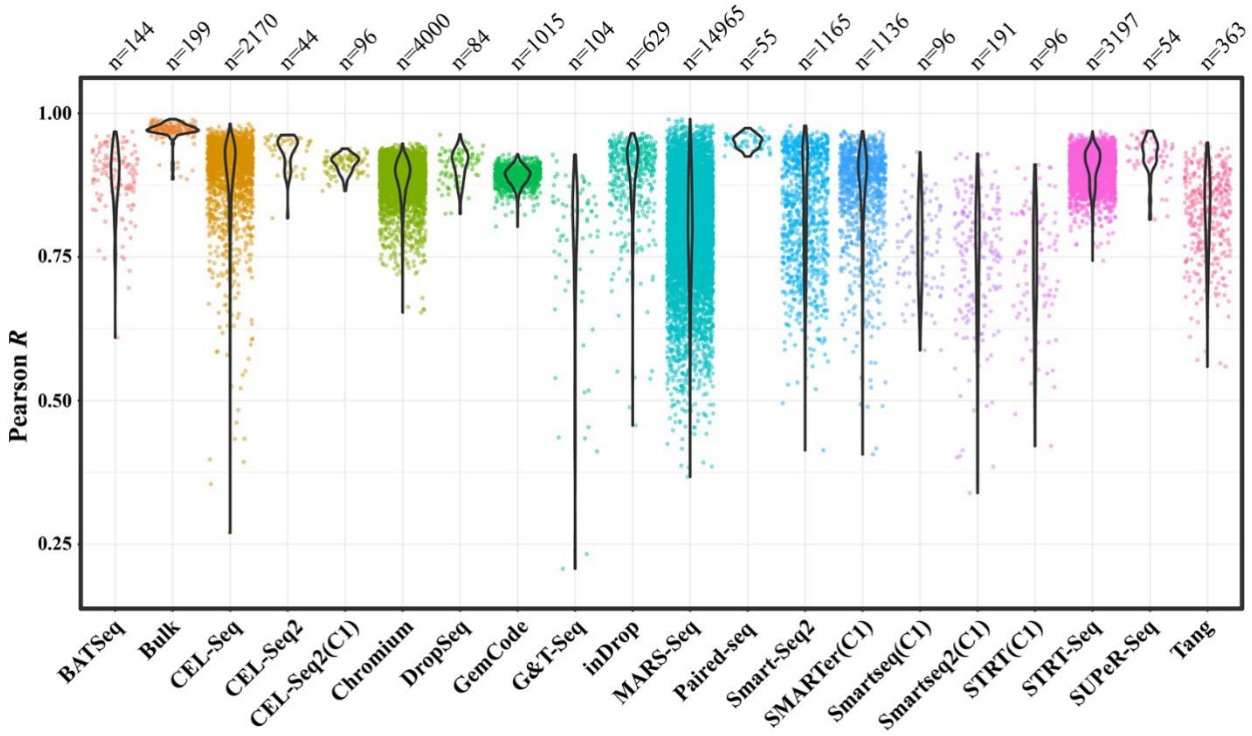

Supplementary Figure 13| Accuracy of different mRNA sequencing methods through ERCC detection. Distributions of Pearson correlations (R) for all samples, stratified by protocol (without accounting for sequencing depth). BAT-seq, barcoded 3′-specific sequencing. n, number of samples. Source data are provided as a Source Data file.

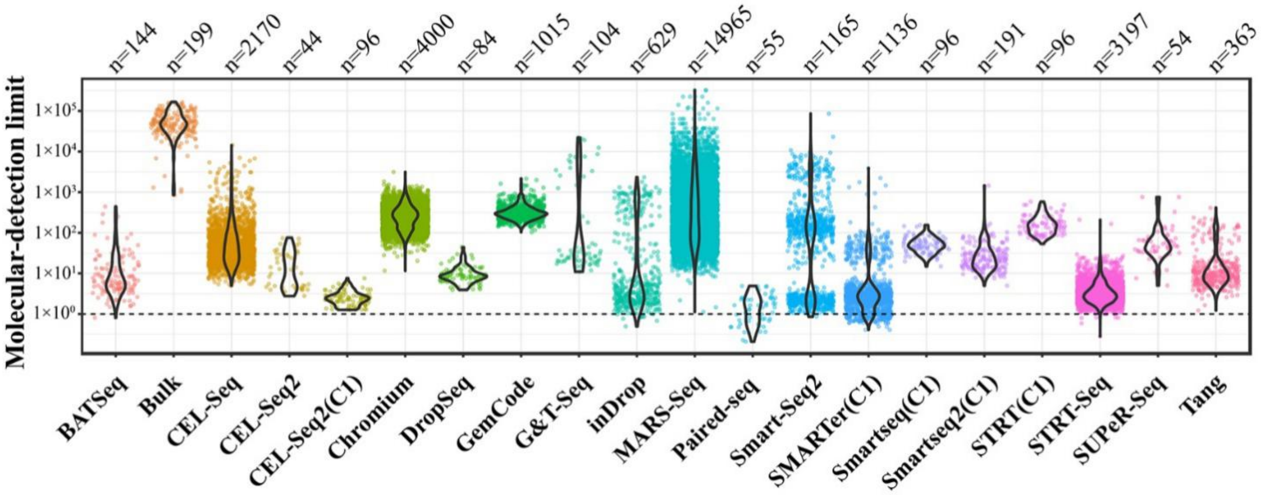

Supplementary Figure 14| Sensitivity of different mRNA sequencing methods through ERCC detection. Distributions of molecular-detection limits for all samples, stratified by protocol (without accounting for sequencing depth). n, number of samples. Source data are provided as a Source Data file.

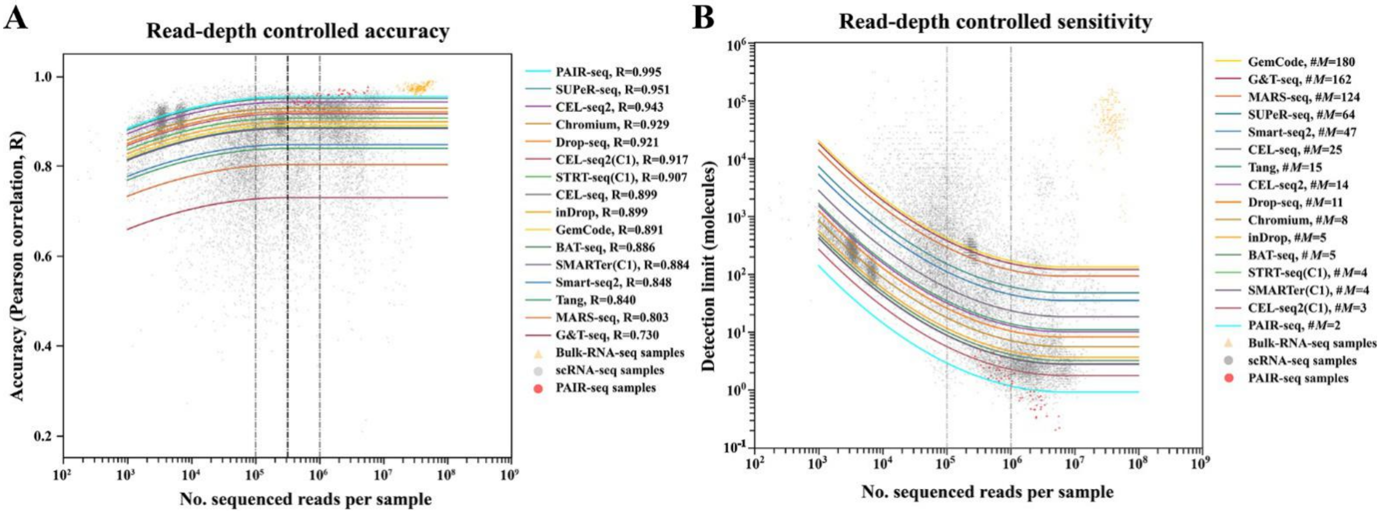

Supplementary Figure 15| Performance metrics in consideration of sequencing depth

(A). Accuracy with a global dependency on sequencing depth indicated the highest accuracy of Paired-seq compared to the other 15 methods.

(B). Sensitivity with a global dependency on sequencing depth indicated the highest sensitivity of Paired-seq compared to the other 15 methods.

Source data are provided as a Source Data file.

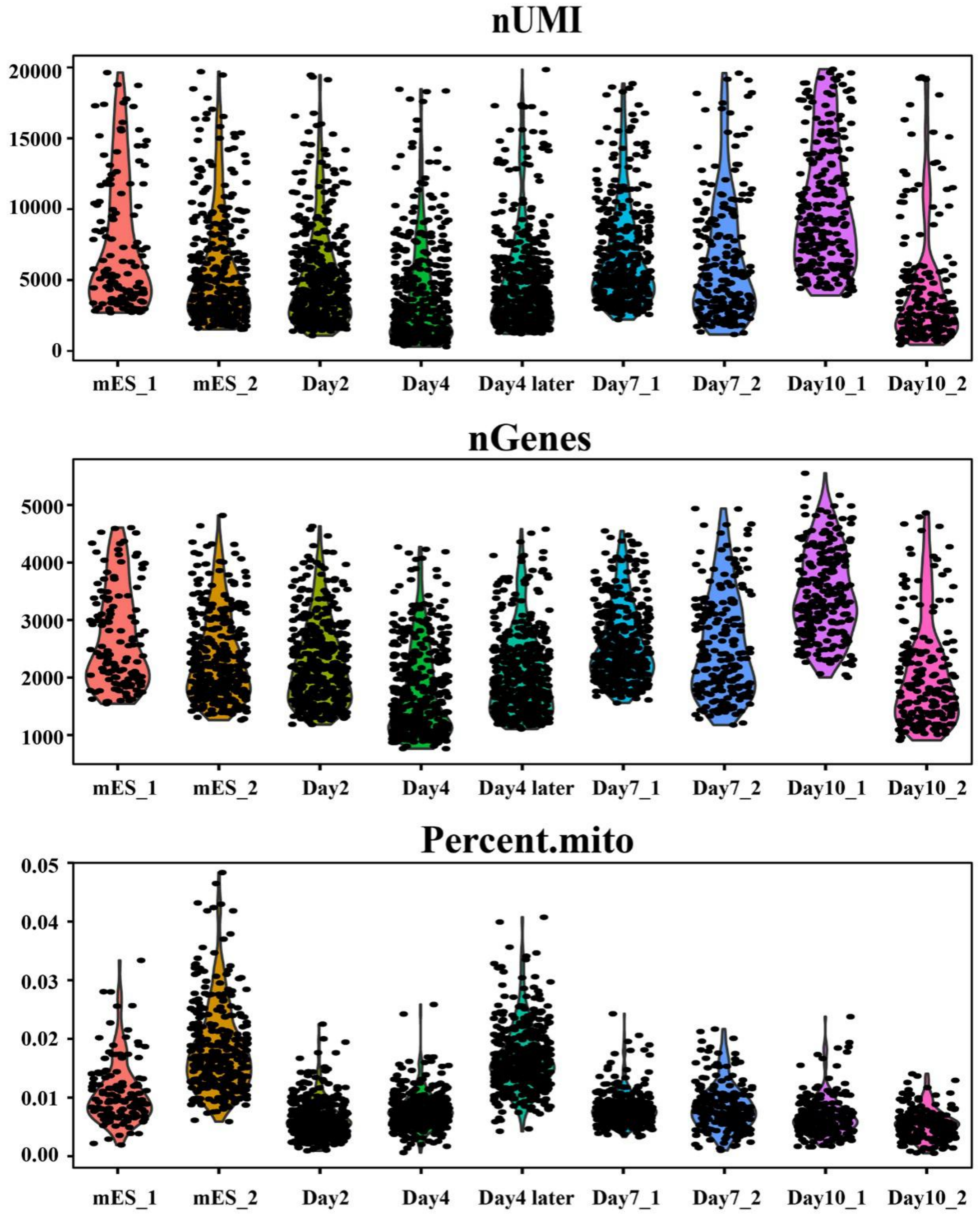

Supplementary Figure 16| Reads mapping quality in mES cells cultured in different days after LIF withdrawal. Source data are provided as a Source Data file.

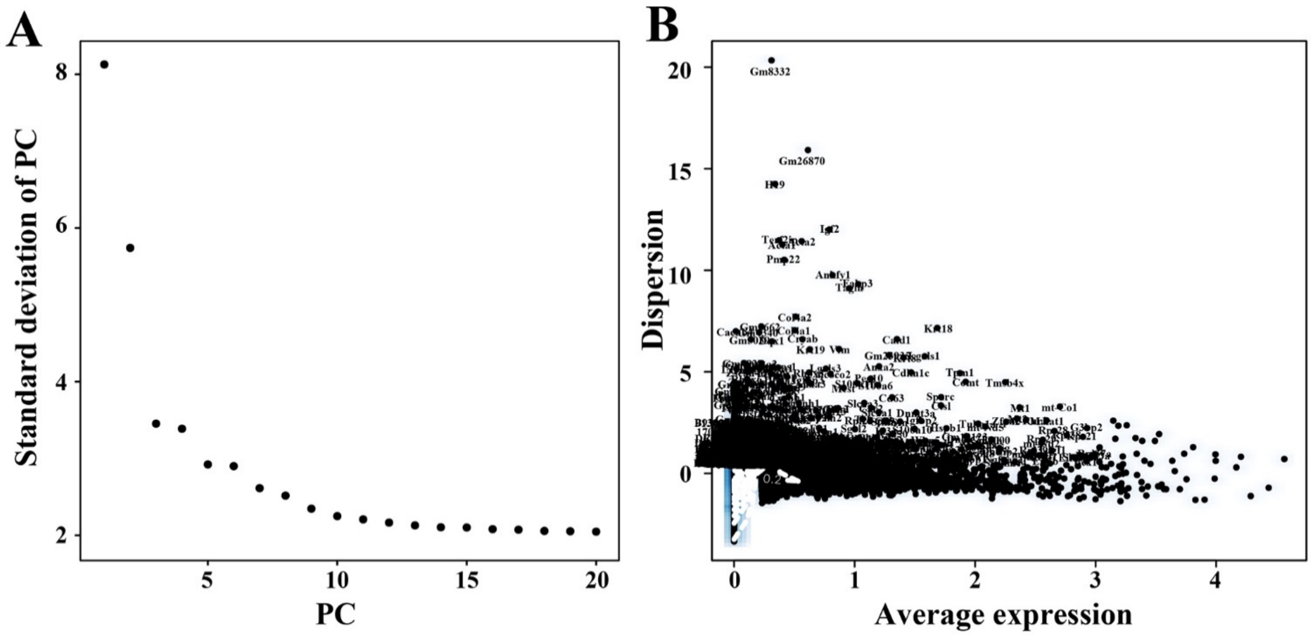

Supplementary Figure 17| Parameters for t-SNE of mES cells

(A) Determination of significant principal components. We choose to look at a plot of the standard deviations of the principle components and draw the cutoff where there is a clear elbow in the graph.

(B) Identification of highly variable genes. Seurat calculates highly variable genes by calculating the average expression and dispersion for each gene. Source data are provided as a Source Data file.

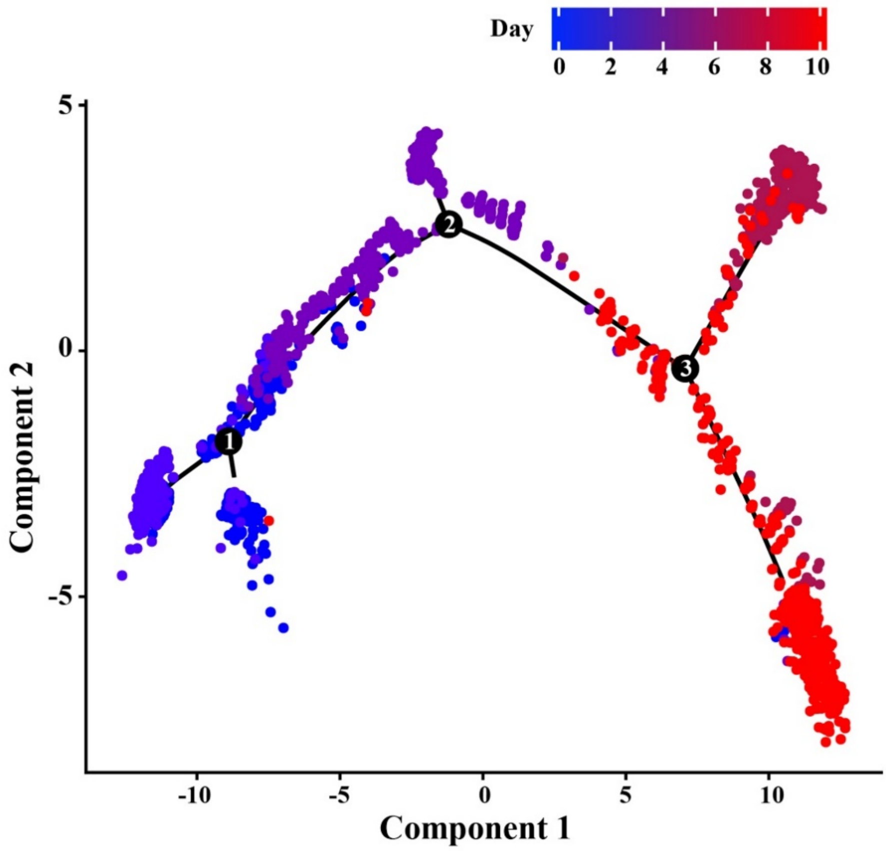

Supplementary Figure 18| Single cell trajectories of differentiated mES cells.

The Monocle (version 2.3.4) package[2] was used to analyze single cell trajectories to discover the differentiation of mES cells along the days. We used differentially expressed genes identified during clusters by Seurat to sort cells in pseudo-time order. 'DDRTree' was applied to reduce dimensions and the minimum spanning tree on cells is plotted and colored by day. Source data are provided as a Source Data file.

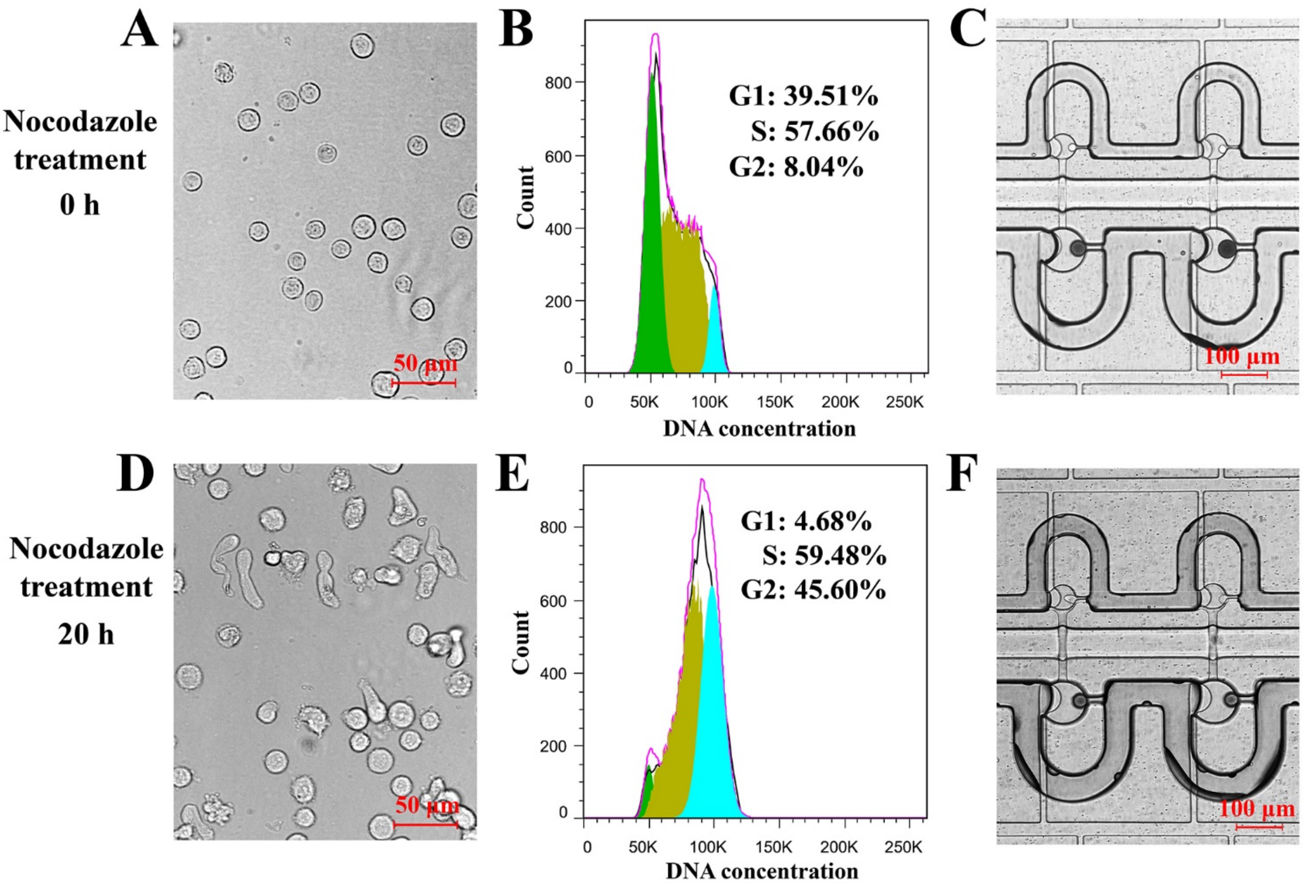

Supplementary Figure 19| *Nocodazole* treatment of K562

Cell cycle analysis by the flow cytometry and morphology characteristic by microscope of 20 h *Nocodazole* treatment on K562 (D-F) and control group (A-C). Scale bar of A and D is 50 μm. Scale bar of C and F is 100 μm. In A, C, D and F, representative results from more than three independent experiments are shown.
Source data are provided as a Source Data file.

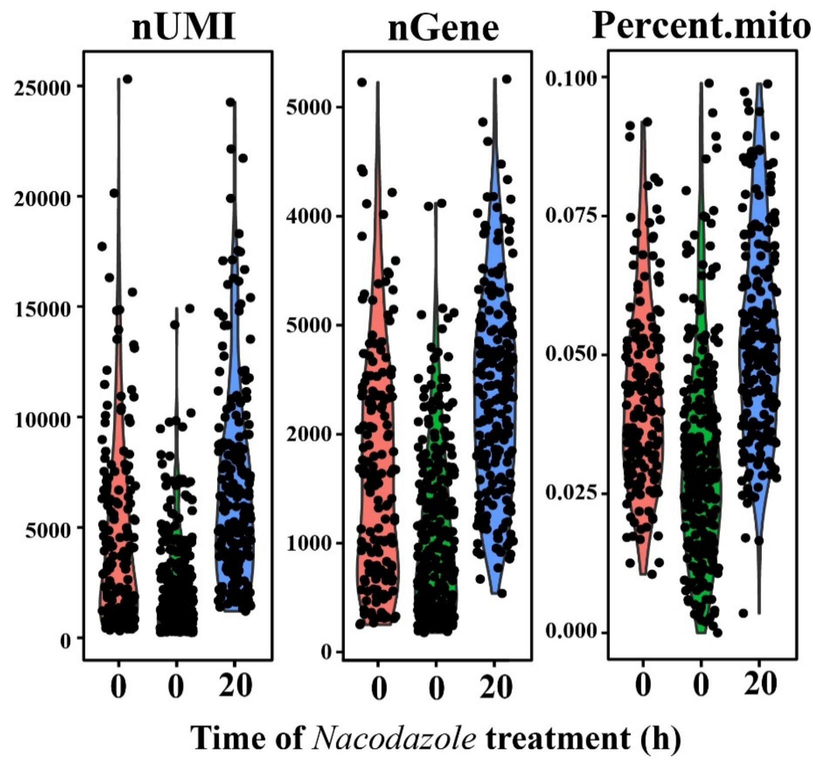

Supplementary Figure 20| Reads mapping quality in *Nocodazole* treated and untreated K562 cells. Source data are provided as a Source Data file.

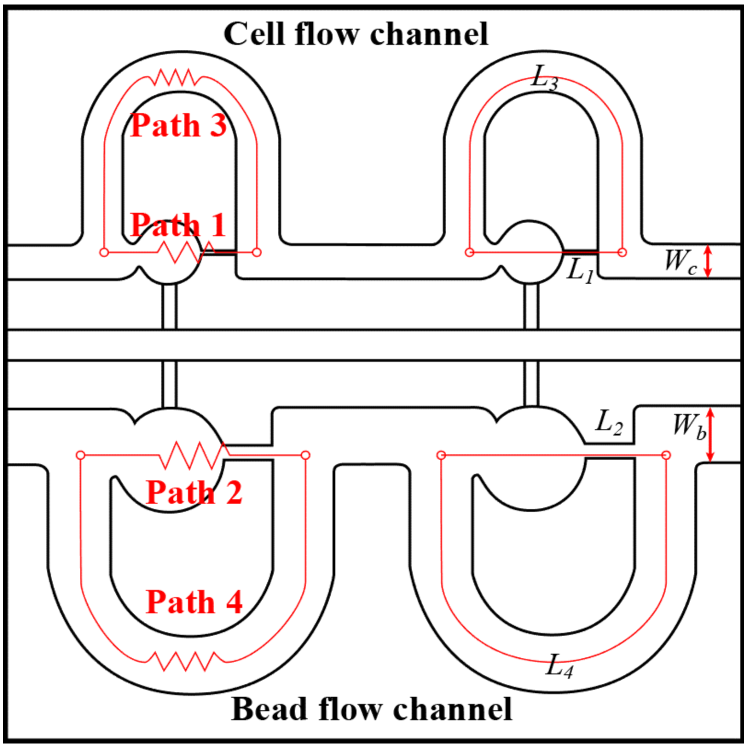

Supplementary Figure 21| Schematic diagram for theoretical calculation of fluid resistance in chip channel.

B. Supplementary Table Legends

Supplementary Table 1. Geometric dimensions of paired unit. All dimensions in μm.

For cells	W_1	L_1	α_1	$C(\alpha_1)$	W_3	L_3	α_3	$C(\alpha_3)$	H_C	Q_1/Q_3
	10.00	24.00	0.42	64.88	35.00	420.00	0.08	86.36	25.00	1.60
For beads	W_2	L_2	α_2	$C(\alpha_2)$	W	L_4	α_4	$C(\alpha_4)$	H_B	Q_2/Q_4
	18.00	32.60	0.55	60.93	48.30	510.00	0.09	85.22	46.00	2.46

Supplementary Table 2. Oligos used in Paired-seq

Name	Sequence (5' to 3')
**Barcoded beads**	TTTTTTTAAGCAGTGGTATCAACGCAGAGTACJJJJJJJJJJJJNNNNNNNNVTTTTTTTTTTTTTTTTTTTTTTTTTTTTTT
**TSO**	AAGCAGTGGTATCAACGCAGAGTGAATrGrGrG
**custom primer P5**	AATGATACGGCGACCACCGAGATCTACACGCCTGTCCGCGGAAGCAGTGGTATCAACGCAGAGT*A*C
**Custom read 1**	GCCTGTCCGCGGAAGCAGTGGTATCAACGCAGAGTAC
**Target DNA**	AAAAAAAAAAAAAAAAAAAA - FITC
**Random DNA**	ACGTTACTCGAATCAGGCGT - FITC

C. Step by Step Protocol for Paired-seq:

1. Reagents:
1) DPBS, no calcium, no magnesium (ThermoFisher Scientific, Cat # 14190250)
2) Water-DEPC Treated Water (Sangon Biotech, Cat# B501005)
3) Alginic acid sodium salt (Sigma-Aldrich, Cat # 180947)
4) Pluronic F-68 Non-ionic Surfactant (ThermoFisher Scientific, Cat#24040032)
5) N-Lauroylsarcosine sodium salt solution 20%, for molecular biology (Sigma-Aldrich, Cat # L7414)
6) Fetal Bovine Serum (ThermoFisher Scientific, Cat# 12483020)
7) Dulbecco's Modified Eagle Medium (ThermoFisher Scientific, Cat # 11965092)
8) 0.25% trypsin-EDTA (ThermoFisher Scientific, Cat # 25200072)
9) TritonX-100 (Sangon Biotech, Cat # A110694)
10) 0.5 M EDTA (ThermoFisher Scientific, Cat # AM9262)
11) 1M Tris pH 7.5 (ThermoFisher Scientific, Cat # 15567027)
12) UltraPure 1M Tris-HCI, pH 8.0 (ThermoFisher Scientific, Cat # 15568025)
13) DTT Solution (2M) (Sangon Biotech, Cat# B645939)
14) 10% SDS solution (ThermoFisher Scientific, Cat # AM9822)
15) Tween 20 solution (Sigma-Aldrich, Cat # P9416)
16) High Pure dNTPs (10 mM) (TransGen Biotech , Cat #AD101-12)
17) RNase Inhibitor (ThermoFisher Scientific, Cat # N8080119)
18) Maxima H Minus Reverse Transcriptase (ThermoFisher Scientific, Cat # EP0753)
19) Exonuclease I (E. coli) (NEB, Cat # M0293L)
20) 2x Kapa Biosystems HiFi Hotstart Readymix (Kapa Biosystems, Cat # KK2601)
21) TruePrep DNA Library Prep Kit V2 for Illumina (Vazyme Biotech, Cat #TD503)
22) VAHTS DNA Clean Beads (Vazyme Biotech, Cat #N411-02)
23) Equalbit dsDNA HS Assay Kit (Vazyme Biotech, Cat # EQ111-02)

2. Primers:
1) Barcoded bead, sequence (5' to 3'): Toyopearl-linker-beads-TTTTTTTAAGCAGTGGTATCAACGCAGAGTAC**JJJJJJJJJJJJ**NNNNNNNNVT(30); **J**: split and pool (twelve); N: degenerated bases (eight); V: (Equimolar ratio of A:C:G) (Chemgenes, Cat # Macosko-2011-10(V+))
2) Template Switch Oligo (5' to 3'): AAGCAGTGGTATCAACGCAGAGTGAATrGrGrG (Sangon Biotech, custom DNA oligo, rG: RNA, HPLC purified)
3) SMART PCR primer (5' to 3'): AAGCAGTGGTATCAACGCAGAGT (Sangon Biotech, custom DNA oligo, standard desalting)
4) P5-PCR hybrid oligo (5' to 3'): AATGATACGGCGACCACCGAGATCTACACGCCTGTCCGCGGAAGCAGTGGTATCAACGCAGAGT*A*C, (Sangon Biotech, custom DNA oligo)
5) Custom Read1 primer (5' to 3'): GCCTGTCCGCGGAAGCAGTGGTATCAACGCAGAGTAC (Sangon Biotech, custom DNA oligo, standard desalting)

3. Consumables:
1) SARTORIUS SFCA 5UM Syringe Filters for sodium alginate solution (SARTORIUS, Cat # 17594-Q)

2) 40 μm cell strainers for beads (PluriSelect, Cat #43-50040-51)
3) 1 mL syringe (BD, Cat # 300841)
4) 2 mL syringe (BD, Cat # 301941)
5) Teflon capillary tube (aixinfu, 0.51 mm inner diameter and 0.90 mm outer diameter)
6) Flea magnet (VP Scientific, cat # 782N-6- 150)
7) 1.5 mL micro-centrifuge tube (Axygen, Cat # 36018934)
8) 0.2 mL micro-centrifuge tube (Axygen, Cat #32818302)
9) 50 mL centrifuge tube (Wantong medical, Cat #WTH04)

4. Special equipment:

1) Microfluidic chip (see CAD file). The unit in the CAD provided is 1 unit = 1 μm; channel depth for beads capture is 46 μm and for cell capture is 36 μm.
2) Microfluidic setup Paired-seq: 6 micro-syringe pumps and a tank of compressed air and associated tube connected to the chip.
3) Inverted microscope

5. Protocol:

5.1. **Beads preparation**:

1) Commercial barcoded beads (Chemgenes, Cat # Macosko-2011-10(V+)) are washed twice with 30 mL of TE/TW (10 mM Tris pH 8.0, 1 mM EDTA, 0.01% Tween), re-suspended in 10 mL TE/TW, and then pass through a 40 μm cell strainer (PluriSelect, Cat # 43-50040-51) into a 50 mL Falcon tube as previously described in Drop-seq protocol. Beads smaller than 40 μm are isolated and placed at 4℃ for long-term storage.
2) Before experiments, about 1000 barcoded beads are transferred into a 0.2 mL micro-centrifuge tube (Axygen, Cat #32818302). Remove the supernatant and re-suspend the barcoded beads in 10 μL 2% sodium alga acid solution with 0.2% Triton X-100. For subsequent beads injection and capture in Paired-seq chip, the suspension is absorbed to the tip of a Teflon capillary connected with the 1 mL syringe which is preset with 300 μL 1×DPBS. There is a section of 0.5-cm length of air between the barcoded beads suspension and 1×DPBS to prevent the solution from contaminating with each other.

5.2 **Cell culture and preservation**:

Cell lines are cultured according to ATCC’s instructions. Cells are treated by 0.25% trypsin-EDTA (ThermoFisher Scientific, Cat # 25200072), washed twice with 1x DPBS re-suspended with a final concentration of 300-500/μL in the suspension buffer (0.2% Pluronic F-68 (ThermoFisher Scientific, Cat#24040032), 4% FBS, 1× DPBS). Absorb about 3-4 μL cell suspension to the tip of a Teflon capillary connected with the 1 mL syringe for subsequent cells injection and capture in Paired-seq chip. The syringe is preset with 300 μL 1×DPBS, and there is a section of 0.5-cm length of air between the cell suspension and 1×DPBS to prevent the solution from contaminating with each other.

5.3. **Operation on Paired-seq chip**:

1) Prepare three individual micro-syringe pumps with three 2 mL syringes with 1 mL ddH_2O for control layer. One syringe is connected with blocking pump channel and the other two are connected with driving pump channels. By controlling the volume of ddH_2O injected into the closed loop circuit, the pressure in the control layer channels could be changed.
2) Inject 3 μL ddH_2O into the three closed loop circuits to remove air and fill the channels with ddH_2O.

3) Prepare two 1 mL syringes with 300 μL 1×DPBS on two individual micro-syringe pumps for rinsing the cell and buffer channel.
4) **Single beads capture.** Set the syringe with barcoded beads suspension on an individual micro-syringe pump. Flow rate for bead inlet, cell inlet and 1×DPBS buffer inlet are 0.2 mL h^{-1}, 0.06 mL h^{-1} and 0.06 mL h^{-1}, respectively. After finishing the bead capture, the bead channel is washed with 1× DPBS to replace sodium alga acid and Triton X-100 while the driving pump for bead is activated to prevent bead escape. Beads arraying time is about 20 min. The connection between Teflon tube and chip can refer to *Supplementary Figure 1* and Figure 1 below.
5) **Single cells capture and pairing with single beads.** Change the syringe connected with cell inlet to pre-prepared syringe (prepare in step 5.2) for single cells capture. Flow rate for bead inlet, cell inlet and 1×DPBS buffer inlet are 0.03 mL h^{-1}, 0.015 mL h^{-1} and 0.015 mL h^{-1}, respectively. **Before cells injection, we must ensure that the isolation valve works well and that all connections channel are disconnected until the paired droplets are formed.**
6) Prepare the lysis buffer (160 mM Tris pH 7.5 (ThermoFisher Scientific, Cat # 15567027) 0.16% Sarkosyl (Sigma-Aldrich, Cat # L7414), 16 mM EDTA (ThermoFisher Scientific, Cat # AM9262), 0.5 U μL-1 RNase Inhibitor (ThermoFisher Scientific, Cat # N8080119), and 0.12% F68(ThermoFisher Scientific, Cat#24040032)) and inject into the bead channel with 5 μL. The flow rate is 0.03 mL h^{-1}.
7) **Droplets generation.** The cell and bead inlets are unplugged and both cell and bead outlets are blocked. Compressed air is reversely injected into the channels, generating water-in-gas droplets, which contain single beads and single cells at a gas flow rate of 0.02 L min^{-1}. Time for droplets generation is about 5 min.
8) **Parallel paired droplets mixing.** Turn off the blocking valve to enable solution exchange between the paired chambers through the connection channel. By alternately increasing and decreasing the pressure of driving pump to activate the driving pump for cells and the driving pump for beads by control the stepper motor of the micro-syringe pump moves forward or backward, solutions in two chambers can be easily transferred back and forth, thus allowing efficient cell lysis and mRNA capture.
9) Turn on the blocking valve and wash the cell channel and bead channel with 1× DPBS independently.
10) **Reverse transcription**. Inject 10 μL reverse transcription mix (1x RT buffer, 1 mM dNTPs (TransGen Biotech , Cat #AD101-12), 1 U μL^{-1} RNase Inhibitor (ThermoFisher Scientific, Cat # N8080119), 2.5 μM Template_Switch_Oligo, and 10 U μL^{-1} Maxima H-RT (ThermoFisher Scientific, Cat # EP0753) in Paired-seq chip at a flow rate of 0.2 mL $h^{-1}$for both cell and bead channel. All the inlets and outlets are sealed with Teflon tape and sealing film. Put the sealed Paired-seq chip in a 50 mL centrifuge tube with 20 mL ddH_2O. Incubate the 50 mL centrifuge tube with Paired-seq chip at room temperature for 30 min followed by 42 ℃ for 90 min (Figure 2-5).
11) **Post RT wash, Exonuclease I treatment.** 10 μL TE-SDS ((10 mM Tris pH 8.0, 1 mM EDTA, 0.5% SDS (ThermoFisher Scientific, Cat # AM9822)), 10 μL TE/TW, and 10 μL TE (10 mM Tris pH 8.0) is successively injected into beads channel of Paired-seq chip in turn with driving pump activated to trap the barcoded beads in the original position. Then 20 μL Exonuclease I mix (1x Exonuclease I Buffer and 1 U μL^{-1} Exonuclease I (NEB, Cat # M0293L)) is injected into beads and cells channel. There are a section of 0.5-cm length of air between the TE-SDS, TE/TW, TE and Exonuclease I mix to prevent the solution from contaminating with each other. We just need to inject one time for four different kinds of reagent. All the inlets and outlets are sealed with Teflon tape and sealing film after the channel is filled with Exonuclease I mix. Put the sealed Paired-seq chip in a 50 mL centrifuge tube

with 20 mL ddH_2O. Incubate the 50 mL centrifuge tube with Paired-seq chip at 37℃ for 45 min (Figure 6).

12) **Barcoded beads Recovery.** 10 μL TE/SDS, 10 μL TE/TW, 10 μL ddH_2O is successively injected into beads channel of Paired-seq chip to wash the barcoded beads and remove Exonuclease I mix (same operation with 11)). After reducing the pressure of the bead driving pump, a high speed of 0.3 mL h^{-1} ddH_2O is introduced to push the advance of beads, making them gather at the end of channel. With the help of water phase flow and gas phase flow in the direction of bead outlet, the barcoded beads can be collected from outlet into a 0.2-mL tube without remnant.
13) **cDNA amplification**. Remove all supernatant and resuspend all collected beads from one single chip with PCR mix (24.6 μL H_2O, 0.4 μL 100 μM SMART PCR primer and 25 μL 2x Kapa Biosystems HiFi Hotstart Readymix (Kapa Biosystems, Cat # KK2601)) for PCR amplification. The PCR program is as follows: 95 ℃ for 3 min; and then 4 cycles of: 98 ℃ for 20 s, 65 ℃ for 45 s, 72 ℃ for 3 min; then 10 cycles of 98 ℃ for 20 s, 67 ℃ for 20 s, 72 ℃ for 3 min; then a final extension step of 5 min. The PCR products are purified using 0.6x VAHTS DNA Clean Beads (Vazyme Biotech, Cat #N411-02) according to the manufacturer's instructions twice, and eluted with 11 μL H_2O. The concentration of the purified products is quantified by qubit3.0.
14) **Library preparation.** The 3'-end enriched sequencing library is prepared using a TruePrep DNA Library Prep Kit V2 for Illumina (Vazyme Biotech, Cat #TD503), according to the manufacturer's instructions, except that the custom primer P5 is used in place of the kit's oligos. The samples are then amplified as follows: 72 ℃ for 3 min, 98 ℃ for 30 s; and 12 cycles of: 98 ℃ for 15 s, 55℃ for 30 s, 72 ℃ for 30 s; then a final extension step of 5 min. The 3'-end enriched library products are purified using 0.6x VAHTS DNA Clean Beads (Vazyme Biotech, Cat #N411-02), and eluted in 11 μL H_2O. The concentration is quantified by qubit3.0. The fragment size of the 3'-end enriched sequencing library is analyzed by Qsep-100, and the average size is between 450 and 650 bp.
15) The libraries are sequenced on the Illumina Nextseq 550 according to the manufacturer's instructions, except that Custom read 1 is used for priming of read 1. Read 1 is 21 bp; read 2 is 60 bp for all the experiments.

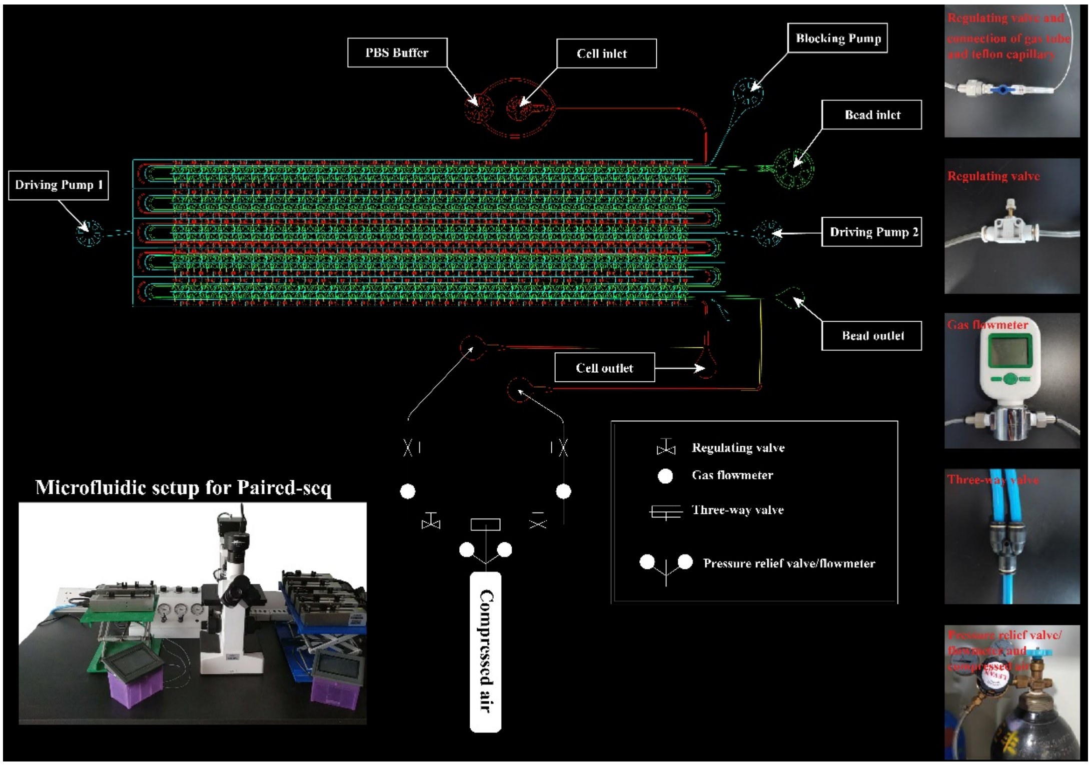

Figure 1. Microfluidic setup and tube connection for Paired-seq.

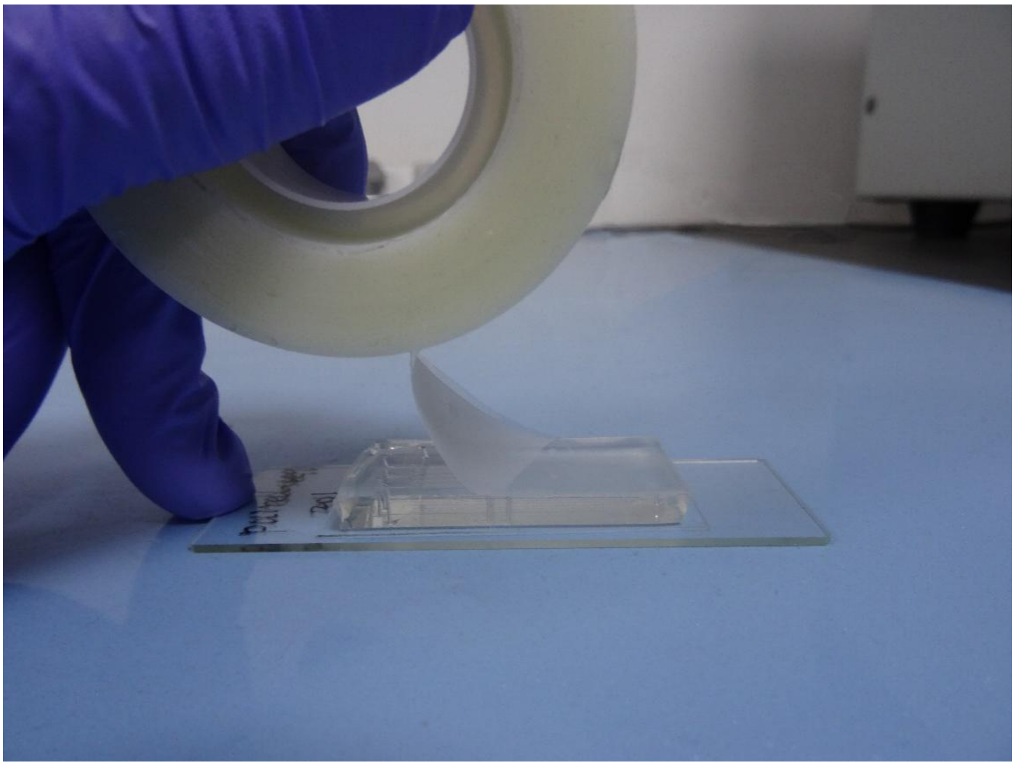

Figure 2. Seal all inlets and outlets of the chip with Teflon tape

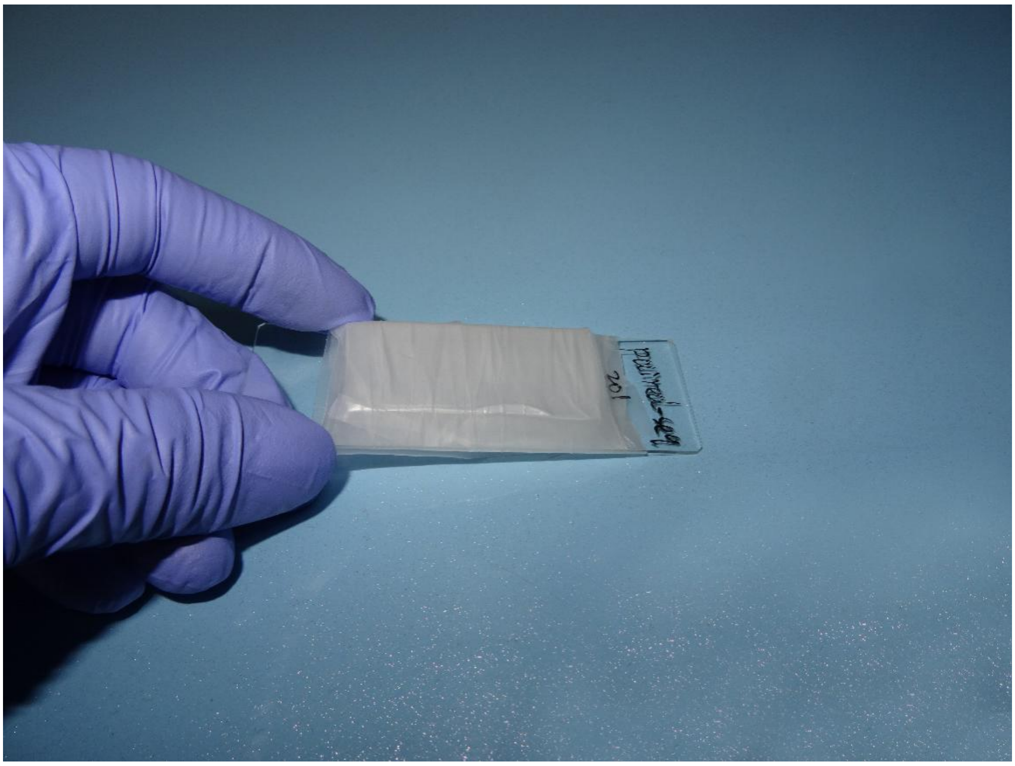

Figure 3. Wrap the chip in a sealing film

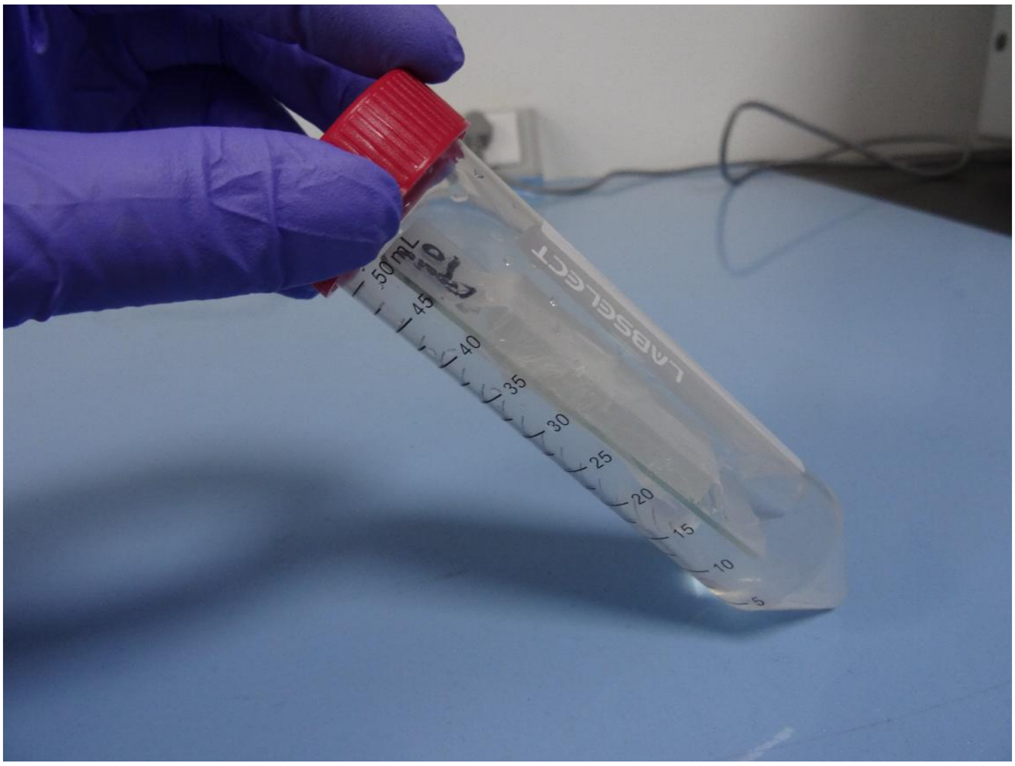

Figure 4. Put Paired-seq chip in a 50 mL centrifuge tube with 20 mL ddH_2O in it for incubation.

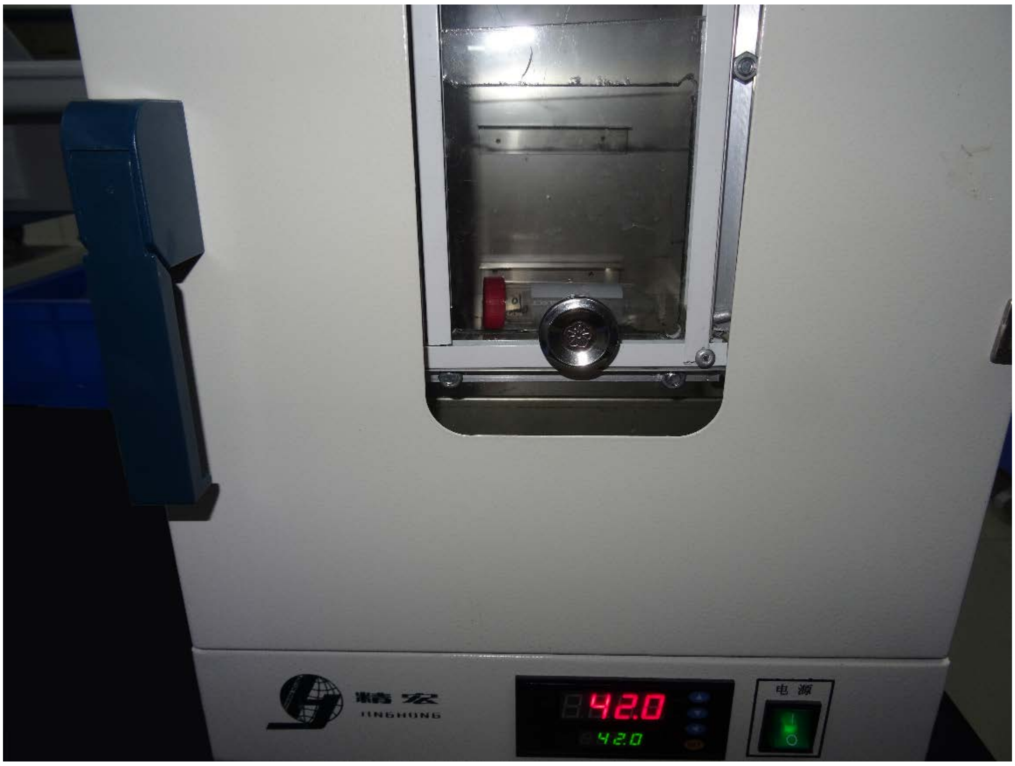

Figure 5. Reverse transcription

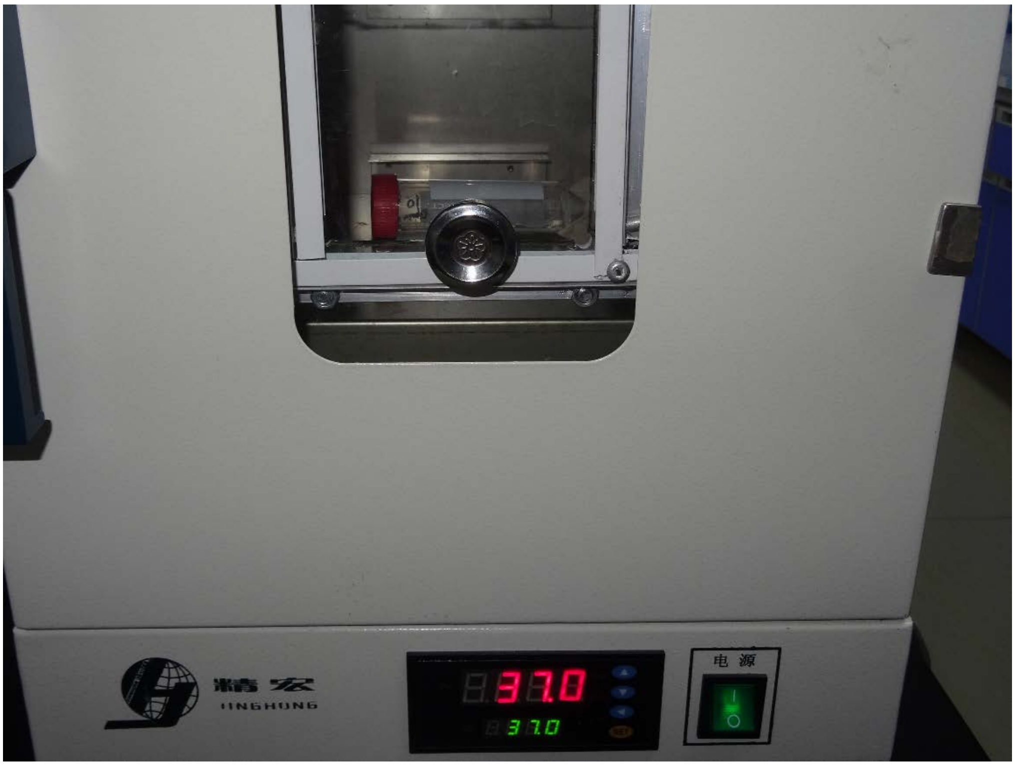

Figure 6. Exonuclease I treatment

D. Supplementary References

1. R. M. S. de Araujo, Y. Oba, K. Moriyama. Identification of genes related to mechanical stress in human periodontal ligament cells using microarray analysis. *J Periodont Res.* **42**, 15-22 (2007).

2. Satija, R. et al. Spatial reconstruction of single-cell gene expression data. *Nat. Biotechnol* **33**, 495-502 (2015).
