# Supplementary material for: Highly parallel and efficient single cell mRNA sequencing with paired picoliter chambers
Source: Nat Commun. 2020 Apr 30;11:2118. doi: 10.1038/s41467-020-15765-0 (PMC7193604; doi:10.1038/s41467-020-15765-0)
Supplement: Supplementary file 3 — Reporting Summary [file 41467_2020_15765_MOESM3_ESM.pdf]

nature research

Corresponding author(s): Chaoyong Yang

Last updated by author(s): Mar 20, 2020

Reporting Summary

Nature Research wishes to improve the reproducibility of the work that we publish. This form provides structure for consistency and transparency in reporting. For further information on Nature Research policies, see Authors & Referees and the Editorial Policy Checklist .

Statistics

For all statistical analyses, confirm that the following items are present in the figure legend, table legend, main text, or Methods section.

n/a	Confirmed	
☐	☒	The exact sample size (*n*) for each experimental group/condition, given as a discrete number and unit of measurement
☐	☒	A statement on whether measurements were taken from distinct samples or whether the same sample was measured repeatedly
☐	☒	The statistical test(s) used AND whether they are one- or two-sided<br>*Only common tests should be described solely by name; describe more complex techniques in the Methods section.*
☐	☒	A description of all covariates tested
☐	☒	A description of any assumptions or corrections, such as tests of normality and adjustment for multiple comparisons
☐	☒	A full description of the statistical parameters including central tendency (e.g. means) or other basic estimates (e.g. regression coefficient) AND variation (e.g. standard deviation) or associated estimates of uncertainty (e.g. confidence intervals)
☐	☒	For null hypothesis testing, the test statistic (e.g. *F*, *t*, *r*) with confidence intervals, effect sizes, degrees of freedom and *P* value noted<br>*Give P values as exact values whenever suitable.*
☒	☐	For Bayesian analysis, information on the choice of priors and Markov chain Monte Carlo settings
☐	☒	For hierarchical and complex designs, identification of the appropriate level for tests and full reporting of outcomes
☐	☒	Estimates of effect sizes (e.g. Cohen's *d*, Pearson's *r*), indicating how they were calculated

Our web collection on statistics for biologists contains articles on many of the points above.

Software and code

Policy information about availability of computer code

Data collection	NIS-Elements Basic Research for microscopy imaging
Data analysis	scRNA-seq data was analyzed using the 'Seurat' R package (V2.3.4), 'monocle' R package (V2.6.4), R (V3.4.4).<br>scRNA-seq expression library FASTQs were pre-processed using python (V3.7.0).<br>scRNA-seq gene name were tagged using Drop-seq software TagReadWithGeneExon (V1.13)<br>scRNA-seq FASTQs were aligned using STAR (V2.5) with references hg19 and mm10

For manuscripts utilizing custom algorithms or software that are central to the research but not yet described in published literature, software must be made available to editors/reviewers. We strongly encourage code deposition in a community repository (e.g. GitHub). See the Nature Research guidelines for submitting code & software for further information.

Data

Policy information about availability of data

All manuscripts must include a data availability statement. This statement should provide the following information, where applicable:

- Accession codes, unique identifiers, or web links for publicly available datasets
- A list of figures that have associated raw data
- A description of any restrictions on data availability

The sequencing data presented in this paper have been deposited in the Sequence Read Archive (SRA) under BioProject accession number PRJNA578456 [https://trace.ncbi.nlm.nih.gov/Traces/sra/?study=SRP226387]. SRA, PRJNA305381; GEO: GSE75790, etc. were referenced in the (supplementary dataset) manuscript. Relevant count and metadata for each main text and supplemental figure are available in the Supplementary Materials.

Field-specific reporting

Please select the one below that is the best fit for your research. If you are not sure, read the appropriate sections before making your selection.

[x] Life sciences [ ] Behavioural & social sciences [ ] Ecological, evolutionary & environmental sciences

For a reference copy of the document with all sections, see nature.com/documents/nr-reporting-summary-flat.pdf

Life sciences study design

All studies must disclose on these points even when the disclosure is negative.

Sample size	For technical characteristic, we used 3 biological replicates for each outcome. This is a widely used standard for statistic analysis.<br>For single-cell RNAseq, we isolated cells from individual cell preparations for each timepoint. We aimed to collect as many cells as possible for analysis, but decided to randomly down-sample each timepoint to 100 cells to balance the samples for easier statistical analysis and cleaner visualization of the data. For ERCC sample, we isolated 55 samples for highly deep sequencing that was comparable to analysis in other platforms.
Data exclusions	No data was excluded from the analysis. Filtering and quality control of single cell RNA-seq data is described in the Methods.
Replication	All experimental steps are detailed in the manuscript to ensure replication. All data analyses are available online in reproducible format. We have not attempted study replication.
Randomization	The study is exploratory and descriptive to demonstrate the feasibility and efficiency of a new scRNA-seq method, and no case control comparisons were performed, so no randomization was considered.
Blinding	Not applicable, the manuscript concerns a new method for preparation of single cell RNA sequencing sample.

Reporting for specific materials, systems and methods

We require information from authors about some types of materials, experimental systems and methods used in many studies. Here, indicate whether each material, system or method listed is relevant to your study. If you are not sure if a list item applies to your research, read the appropriate section before selecting a response.

Materials & experimental systems

n/a	Involved in the study	
[x]	[ ]	Antibodies
[ ]	[x]	Eukaryotic cell lines
[x]	[ ]	Palaeontology
[x]	[ ]	Animals and other organisms
[x]	[ ]	Human research participants
[x]	[ ]	Clinical data

Methods

n/a	Involved in the study	
[x]	[ ]	ChIP-seq
[ ]	[x]	Flow cytometry
[x]	[ ]	MRI-based neuroimaging

Eukaryotic cell lines

Policy information about cell lines

Cell line source(s)	NIH 3T3: ATCC CRL-1658, purchased from National Infrastructure of Cell Line Resource.<br>K562: ATCC CCL-243, purchased from National Infrastructure of Cell Line Resource.<br>J1 mouse embryonic stem cell (J1 mES cell): derived from the mouse 129 s4 / SvJae strains called J1. They were kindly provided by Stem Cell Bank, Chinese Academy of Sciences.
Authentication	NIH 3T3/K562/J1 mES: Cells were authenticated at their source (e.g., ATCC) prior to acquisition, but no extra authentication was employed in this study. Single-cell gene expression profiles match expectations from literature-supported marker genes for each cell line.
Mycoplasma contamination	N/A
Commonly misidentified lines<br>(See ICLAC register)	N/A

Flow Cytometry

Plots

Confirm that:

- [x] The axis labels state the marker and fluorochrome used (e.g. CD4-FITC).
- [x] The axis scales are clearly visible. Include numbers along axes only for bottom left plot of group (a 'group' is an analysis of identical markers).
- [x] All plots are contour plots with outliers or pseudocolor plots.
- [x] A numerical value for number of cells or percentage (with statistics) is provided.

Methodology

Sample preparation	Drug treated cells: K562 cells were cultured in Dulbecco's Modified Eagle's Medium supplemented with 10% Fetal Bovine Serum, 1% Penicillin-streptomycin and 1 nM Nocodazole for 20 h.<br>Control cells: K562 cells were cultured in Dulbecco's Modified Eagle's Medium supplemented with 10% Fetal Bovine Serum, 1% Penicillin-streptomycin for 20 h.<br><br>For flow cytometry analysis, the medium was removed, and the cells were washed for three times with cold 1× PBS buffer and then re-suspended in 400 μL cold 1× PBS and 1100 μL cold fixing solution (100% ethyl alcohol) and stored overnight at 4 ℃. The next day cells were centrifuged to remove the fixing solution, washed three times with 1× PBS and re-suspended in 500 μL 1× PBS. RNase A (ThermoFisher, 20 mg/L) was introduced to remove the interference of RNA at 37 ℃ for 1 h. Then the nuclear DNAs of K562 cells were stained by PI (ThermoFisher, 50 mg/L) in a dark place at 4 ℃ for 1 h. One million K562 cells in total were detected by flow cytometry, with the obvious fluorescence peak corresponding to 2n/4n DNAs, which represented the cell cycle position.
Instrument	BD FACSVerse™
Software	FowJo and modifit
Cell population abundance	The control cells and drug treated cells populations were isolated by gating on fluorescence intensity for staining DNA .~93% control cells were at interphase (G1-S) while ~46% Nocodazole treated cells were at mitosis (G2-M).
Gating strategy	Live cells were distinguished from debris and cell aggregates via FSC-A x SSC-A gating.<br><br>Gating on FSC-A x SSC-A and FSC-A x FSC-W was used to eliminate cell aggregates and ensure the collection of only single cells. Click Sync Wizard and create or edit model then analysis, the cell cycle and percentage of G1, G2 and S will be shown in the plot. The fluorescence intensity for staining DNA of G2 was twice than that of G1.

- [x] Tick this box to confirm that a figure exemplifying the gating strategy is provided in the Supplementary Information.
